# Exploring Computational and Biophysical Tools to Study the Presence of G-Quadruplex Structures: A Promising Therapeutic Solution for Drug-Resistant *Vibrio cholerae*

**DOI:** 10.3389/fgene.2020.00935

**Published:** 2020-09-25

**Authors:** Uma Shankar, Neha Jain, Prativa Majee, Prashant Kodgire, Tarun Kumar Sharma, Amit Kumar

**Affiliations:** ^1^Discipline of Biosciences and Biomedical Engineering, Indian Institute of Technology Indore, Indore, India; ^2^Translational Health Science and Technology Institute, Faridabad, India

**Keywords:** G-quadruplex, multi drug-resistant, *Vibrio cholerae*, therapeutic strategy, gram-negative

## Abstract

*Vibrio cholerae*, a gram-negative bacterium that causes cholera, has already caused seven major pandemics across the world and infects roughly 1.3–4 million people every year. Cholera treatment primarily involves oral rehydration therapy supplemented with antibiotics. But recently, multidrug-resistant strains of *V. cholerae* have emerged. High genomic plasticity further enhances the pathogenesis of this human pathogen. Guanines in DNA or RNA assemble to form G-quadruplex (GQ) structures which have begun to be seen as potential drug targeting sites for different pathogenic bacteria and viruses. In this perspective, we carried out a genome-wide hunt in *V. cholerae* using a bio-informatics approach and observed ∼85 G-quadruplex forming motifs (VC-PGQs) in chromosome I and ∼45 putative G-quadruplexs (PGQs) in chromosome II. Ten putative G-quadruplex forming motifs (VC-PGQs) were selected on the basis of conservation throughout the genus and functional analysis displayed their location in the essential genes encoding bacterial proteins, for example, methyl-accepting chemotaxis protein, orotate phosphoribosyl transferase protein, amidase proteins, etc. The predicted VC-PGQs were validated using different bio-physical techniques, including Nuclear Magnetic Resonance spectroscopy, Circular Dichroism spectroscopy, and electrophoretic mobility shift assay, which demonstrated the formation of highly stable GQ structures in the bacteria. The interaction of these VC-PGQs with the known specific GQ ligand, TMPyP4, was analyzed using ITC and molecular dynamics studies that displayed the stabilization of the VC-PGQs by the GQ ligands and thus represents a potential therapeutic strategy against this enteric pathogen by inhibiting the PGQ harboring gene expression, thereby inhibiting the bacterial growth and virulence. In summary, this study reveals the presence of conserved GQ forming motifs in the *V. cholerae* genome that has the potential to be used to treat the multi-drug resistance problem of the notorious enteric pathogen.

## Introduction

Cholera, one of the common bacterial diseases, has had a catastrophic effect on the human race. The disease has resulted in seven major pandemic situations since 1817, and is known to be endemic in more than 47 countries, especially in developing countries (Hall, 2018). Cholera is caused by a gram-negative, curved-shaped, flagellated bacterium called *Vibrio cholerae* (*V. cholerae*; Reidl and Klose, 2002). A high infectious dose (10^3^–10^8^) and a very fast replication time of 17 min of *V. cholera*e leads to its higher pathogenesis (Hun Yoon and Waters, 2019). Approximately 1.3–4 million people are infected by this bacterium every year, taking the lives of 21,000–143,000 individuals worldwide (Islam et al., 2017). This disease can be treated if early detection and subsequent treatment is done, but the disease causes major concern as it largely impacts young children in the age group of 1–5 years. *V. cholerae* is usually transmitted through contaminated food and water, and as the bacteria enter the human body, it attaches itself into the epithelial membrane and secretes the cholera toxin. The bacterial infection leads to excessive watery diarrhea which results in severe dehydration followed by shock and, in due course, causes death. There are more than 220 serogroups of *V. cholerae* prevailing in the environment, but the O1 serogroup was mainly accountable for the seven pandemics that have occurred so far (Shimada et al., 1994). Out of this, the first six pandemics were caused by the classical biotype of O1 while the last pandemic was a result of the E1 Tor biotype of *V. cholerae* O1 (Cho et al., 2010). Cholera patients are usually supplemented with antibiotics along with oral rehydration therapy to minimize the severity of the disease and maintain the fluid level of the body. But the use of these antibiotics comes with malediction in the form of resistance. The most commonly used antibiotics against the bacterium includes tetracycline, erythromycin, quinolones, sulfonamides, bleomycin, chloramphenicol, and aminoglycosidase, but due to the extraordinary genomic plasticity, around 90% of the recent *V. cholerae* isolates have attained antimicrobial resistance against them (Verma et al., 2019). The contributing factors towards this increasing antibiotic resistance include chromosomal mutations, export of the antibiotics outside the cell through efflux pumps, and attaining genetic resistance through the exchange of plasmids, transposons, SXT elements, and autonomously replicating and integrating plasmids or integrons (Kumar et al., 2009; Kitaoka et al., 2011a; Verma et al., 2019). Integrating conjugative elements (ICE) and superintegrons from closely or distinctly related bacteriums are the major causes of antimicrobial resistance in *V. cholerae* (Verma et al., 2019; Das et al., 2020). Various strategies have been enforced to comabat these MDR strains, including the usage of various efflux pump inhibitors and quorum sensing inhibitors, but the problem remains unsolved (Hema et al., 2016; Lowrence et al., 2019). This multi-drug resistant strains stands as a barrier to the treatment of cholera disease and demands the attention of the scientific community.

**FIGURE 1:**
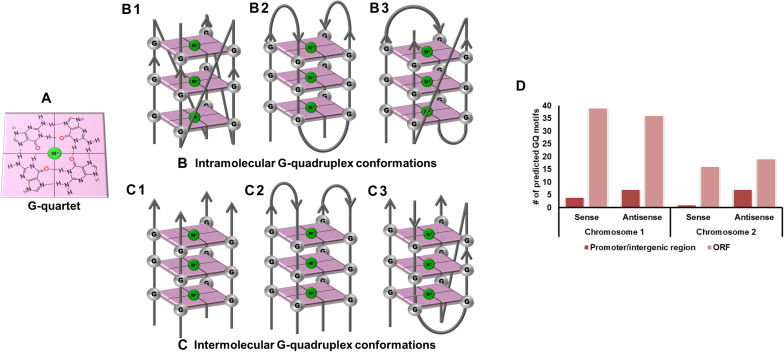
G-quadruplex topologies and GQ in *Vibrio cholerae* reference genome. **(A)** G-tetrad formed by the interaction of 4 guanine residues. Panels **(B,C)** represents the parallel, anti-parallel, and hybrid conformation of G-quadruplex. These G-quadruplex structures have been stabilized by the presence of cations (Green). **(D)** Number of GQ motifs obtained in sense and anti-sense strands of chromosome I (NC_002505.1) and II (NC_002506.1) in the reference genome of *V. cholerae.*

Research in the field of short secondary-structure forming motifs has recently begun to gain momentum. One such type of non-canonical structures which has been extensively studied is G-Quadruplex (GQ) structures. These specialized structures are formed when two or more guanine residues interspersed by some nucleotides self-assemble to form quartet structures (Figure 1). G-quadruplex shows structural polymorphism based on guanine repeats, loop sequences, loop length, physiological conditions, cations available, etc., thus opening up a plethora of motifs for drug designing. The GQ structures have been extensively reported to be found in the human genome, especially in the telomeric end of the chromosome (Patel et al., 2007) and in the promoter regions of the proto-oncogenes like *c-Myc*, *c-Kit*, *bcl2*, and KRAS (Cogoi and Xodo, 2006; Dai et al., 2006a, b; Yang and Hurley, 2006; Huppert and Balasubramanian, 2007). The relevance of GQ structures were found in all the cellular processes, including DNA recombination, RNA transcription, protein translation, and in recombination, which are imperative for a cell’s survival (Kim, 2019). Both the nucleic acids, DNA and RNA, are capable of forming these specialized structures. The GQ structures forming in a specific part of the genome hold a unique role to play; those in the promoter region, for example, modulates the transcription of the genes (Siddiqui-Jain et al., 2002; Rigo et al., 2017; Kong et al., 2018) while those present in the open reading frame (ORF) region affect the replication, transcription, and translation of genes harboring the GQ (Endoh et al., 2013b). Even GQ structures in the untranslated region (UTR) hold significant functions, including miRNA binding (Rouleau et al., 2017), cis-eQTLs, and RNA-binding protein (RBP) interactions (Bugaut and Balasubramanian, 2012; Lee et al., 2020).

Genome mining of GQ forming sequences in different organisms has opened up a new avenue for drug-targeting against the pathogenic organisms (Saranathan and Vivekanandan, 2018). With new reports of GQ structures being involved with different human diseases, development of ligands specifically targeting these GQ structures have also accelerated, and a few have even gone through clinical trials (Yang and Okamoto, 2010; Vy Thi Le et al., 2012; Asamitsu et al., 2018). Various pathogenic organisms, including bacteria, virus, protozoa (Bhartiya et al., 2016), and fungi (Hershman et al., 2008), are reported to bear these unique secondary structures in their genome. For instance, *Mycobacterium tuberculosis* was reported to bear GQ structures in the ORF region of the *espB*, *espK*, and *cyp51* genes (Mishra et al., 2019b) as well as in its gene promoter region (Perrone et al., 2017b). G-quadruplex structures have been shown to be associated with the antigenic variation mechanism of *pilE* gene in *Neisseria gonorrhoeae* (Cahoon and Seifert, 2009; Zhang et al., 2011), *tprK* gene in *Treponema pallidum* (Giacani et al., 2012), and *vlsE* gene in *Borrelia burgdorferi* (Walia and Chaconas, 2013). Interestingly, three highly conserved GQ forming motifs were recognized in the *hsdS*, *recD*, and *pmrA* genes which could act as potential drug targets against pathogenic *Streptococcus pneumoniae* (Mishra et al., 2019a). A recent analysis revealed the presence of GQ motifs in various genes of *Klebsiella pneumoniae* that influenced the gene expression (Shankar et al., 2020). Likewise, several human infecting viruses including Zika (Fleming et al., 2016), Nipah (Majee et al., 2020a), Herpes Simplex virus (Biswas et al., 2016), Human Papillomavirus (Tlučková et al., 2013), and Human Adenovirus (Majee et al., 2020b) are reported to possess potential GQ forming sequences. G-quadruplex sequences in the L gene of Ebola virus and the core gene of Hepatitis C Virus were found to be novel anti-viral drug targets as stabilization of these structures by GQ binding ligands like TMPyP4 [5,10,15,20-Tetrakis-(N-methyl-4-pyridyl) porphine] and PDP negatively regulated the expression of the genes harboring the GQ structures (Wang et al., 2016a, b). Thus, there is an enormous amount of evidence proving the GQ structures to be promising therapeutic targets.

With this perspective, we tried to explore the *V. cholerae* genome for potential GQ forming sequences that could unlock new anti-bacterial strategies for treating cholera. Evaluation of the most recent pandemic causing strain of *V. cholerae*, O1 biovar El Tor str. N16961, revealed the presence of the ten most conserved VC-PGQ (*V. cholerae* – Putative G quadruplex) sequences. As the complete genome of *V. cholerae* is coded by two chromosomes, the large chromosome consists of seven conserved VC-PGQs in the essential bacterial genes, like methyl accepting chemotaxis protein coding gene, *rtxA* gene, GGDEF family protein coding genes, etc., while the other three VC-PGQs are present in the small chromosome. All the ten VC-PGQs of both the chromosomes were found to form stable GQ structures when evaluated through different biophysical techniques like NMR, CD spectroscopy, and EMSA and was also found to have very high binding affinity for the GQ specific ligand, TMPyP4.

## Materials and Methods

### Exploring the Available *Vibrio cholerae* Genome Sequences for PGQ Prediction

All the 68 available complete genome sequences of *V. cholerae* were retrieved from the NCBI genome database^1^. In this study, *V. cholerae* O1 biovar El Tor str. N16961 was used as the reference genome. Each chromosome of the bacteria was individually evaluated for the PGQ prediction. A total of 68 chromosome I and 65 chromosome II of the bacterial strains were checked for the presence of GQ forming sequence through our in-house available GQ prediction tool (Mishra et al., 2016). The PGQ prediction tools make use of the following notion:

$$G{}_{\geq }{}_{3}N{}_{1-20}G{}_{\geq }{}_{3}N{}_{1-20}G{}_{\geq }{}_{3}N{}_{1-20}G{}_{\geq }{}_{3}$$

where G represents to the guanine residues and N represents any of the four nucleotides, including guanine. While the Guanine repeat was set to a minimum of 3, the loop length was set between 1 and 20. The tools scrutinized both the sense and anti-sense strand of the bacterial genome for the PGQ prediction. The PGQ sequences were re-confirmed by using the other online available tool, QGRS Mapper (Kikin et al., 2006). Further, the PGQ sequences were aligned to generate the consensus motifs which depicted the degree of conservation among the nucleotides.

### Determination of Genomic Locations and the Related Functions of the PGQ Sequences

The genomic location and the associated function of the predicted PGQs were determined using the NCBI nucleotide database. The Graphics mode of the GenBank database was further explored for this purpose to analyze whether the PGQ is located in the promoter region, in the ORF, or in the intergenic region.

### Sample Preparation

All the oligonucleotide sequences used in the study were procured from Sigma Aldrich (Bangalore, India), and 100 μM of stock solutions were prepared by adding the required amount of MiliQ water according to the manufacturer’s specification. Then the required dilutions were prepared in Tris-Cl buffer (10mM, pH 7.4) containing 50 mM of either of the four different cations, namely K^+^, Na^+^, Li^+^, and Mg^2+^. The samples were thermally denatured by heating at 92°C for 10 min and then slowly cooled down to room temperature before performing each set of experiments.

### 1D ^1^H Nuclear Magnetic Resonance Studies

The 1D ^1^H nuclear magnetic resonance (NMR) experiments were performed using the AVANCE III 400 MHz Ascend Bruker BioSpin International AG, Switzerland model furnished with the 5 mm broadband inverse (BBI) probe. While Sodium trimethylsilylpropanesulfonate (DSS) was taken as the reference compound, the oligonucleotide sample was prepared in 90/10% H_2_O/D_2_O solution containing 50 mM potassium phosphate buffer at 298 K. The data obtained were further processed and analyzed by using Topspin 4.0 (academic license) software.

### Circular Dichroism (CD) Spectrophotometer Experiments

All the circular dichroism (CD) spectra and CD melting experiments were carried out on the Jasco J-815 Spectropolarimeter (Jasco Hachioji, Tokyo, Japan) accompanied with a temperature controlling Peltier junction. Nitrogen gas was continuously circulated within the instrument to avoid condensation around the cuvette. Quartz cuvette of 1mm path length was used for the experiments. The CD spectra profile for the each PGQ was recorded over the wavelength range of 220–320 nm with a scanning speed of 20 nm/min at 25°C. The final concentration of PGQ DNA was maintained at 20 μM in the 10 mM Tris buffer containing either of the four cations (K^+^, Na^+^, Li^+^, and Mg^2+^). Buffer correction was done for each set of experiments, while each of the spectra was recorded in triplicate. The data obtained from the CD spectrophotometer was then plotted using the SigmaPlot software.

For the CD melting experiments, the temperature range was set between 25 and 98°C with a gradual increase at a rate of 1°C/min. The CD melting plot was collected at a particular wavelength throughout the experiment in the presence of the same Tris-Cl buffer.

### Electrophoretic Mobility Shift Assay

Electrophoretic mobility shift assay (EMSA) was performed with the help of 25% native polyacrylamide gel in the presence of 1X Tris-Borate-EDTA (TBE) buffer. The PGQ sequences were evaluated in four different buffer conditions; therefore, no cation was added into the running TBE buffer (in tank), rather individual oligonucleotide (20 μM concentration) were dissolved in the respective buffer (containing either K^+^, Na^+^, Li^+^, or Mg^2+^) before loading into the gel. Standard GQ forming sequences like *cKit21* was used as the positive control and linear DNA oligonucleotides of the equal length as that of PGQ as the negative control. The gel assays were performed in a Bio-Rad Mini protean Tetra Vertical Electrophoresis unit at 4°C, 90 V. The gels were then soaked in Ethidium Bromide solution for staining and further visualized in ImageQuant LAS4000 (GE Healthcare, Biosciences Ltd., Sweden).

### Isothermal Titration Calorimetry Studies of VC-PGQs With TMPyP4

Isothermal titration calorimetry (ITC) studies between the DNA and ligand was accomplished using the MicroCal iTC200 isothermal titration calorimeter (GE Healthcare, United States) at a constant temperature of 25°C. The oligonucleotide and the ligand solution were prepared in the same buffer, i.e., 1X potassium phosphate buffer (pH 7.4). Total solution of TMPyP4 was titrated into the PGQ containing solution through 21 injections of 1.80 μL each. The duration of each injection was 3.6 s while the spacing between each successive injection was kept at 120 s. The heat of dilution was determined in each set of experiments and further subtracted from the binding isotherms for the purpose of buffer correction. The data obtained were fitted through the “two mode of binding sites” model using the MicroCal Origin software.

### G-Quadruplex Structure Prediction

Two VC-PGQs, VC-I-PGQ-7, and VC-II-PGQ-1 were modeled by exploiting comparative structure modeling using ModeRNA tool (Rother et al., 2011). For structure prediction, PDB ID: 2M27 was used as a template for VC-I-PGQ-7, and PDB ID: 3SC8 was used for VC-II-PGQ-1. The resultant RNA models were converted to DNA using Discovery Studio v4.0. The image processing was performed using Open source PyMOL visualization tool.

### Molecular Docking of GQs With Potassium (K^+^) and TMPyP4

AutoDock tools were used for interaction analysis of GQs with K^+^ and TMPyP4, respectively. TMPyP4 was downloaded from PubChem (CID: 135442972). Receptor (GQ) and ligand (TMPyP4) were prepared using the available AutoDockTools with the MGLTools package and converted to PDBQT files. For VC-I-PGQ-7, a grid was created by using grid points *x* = 44, *y* = 52, *z* = 46 with a grid spacing of 0.61. While for VC-II-PGQ-1, a grid was prepared with grid points *x* = 56, *y* = 52, and *z* = 46 with spacing of 0.61. Docking results were analyzed by running docking log files (.dlg) for the respective complexes.

### Molecular Dynamic Simulation Analysis

Molecular dynamic simulation (MDS) was performed using NAMD Suite (Melo and Bernardi, 2018). Input files for simulation analysis was performed by utilizing CHARMM 36m force fields (Huang et al., 2017) using CHARMM-GUI server (Jo et al., 2008). The modeled GQs were solvated in a rectangular water box with an edge distance of 10.0 with periodic boundary conditions. To neutralize the charges, 0.15 M KCl was added using the Monte–Carlo method of ion placement. The grid was constructed using Particle mesh Ewald – Fast Fourier transformation (PME-FFT). Structures were minimized for 10,000 steps using steepest descent algorithm. Thereafter, the system was equilibrated in isochoric equilibration (NVT) and isobaric conditions (NPT). Nose–Hoover Langevin algorithm was used for pressure control. After this, a production run of 10 ns was performed at constant temperature of 310 K. The trajectory analysis was performed using CPPTraj tool (Roe and Cheatham, 2013).

For analyzing the global fluctuations in the system, root mean square deviation (RMSD) was calculated. For RMSD calculation, the first frame (initial structure at TS = 0 ps) was used as a reference, and rest frames in the simulation trajectory were compared. For global packaging analysis, radius of gyrations (Rg) was generated for all the time frames.

## Results

### Mining of Conserved PGQ Sequences From the *Vibrio cholerae* Genome

*Vibrio cholerae* interestingly possesses its complete genome in two circular chromosomes, a large chromosome bearing most of the essential genes required for cell growth and pathogenicity and a small chromosome that contains most of the hypothetical protein coding genes and the supporting genes helping the bacteria to survive different environmental conditions. This bipartite genome was evaluated for the probable GQ forming sequences and, due to the bi-directional mode of replication, both sense and anti-sense strands were taken into consideration. In GQ structure formation, G-tract and loop length plays a major role and it is considered that a minimum G-tract of 3 and loop length in the range of 1–20 nucleotides can form stable GQ conformation *in vitro* and *in vivo*. Therefore, both the chromosomes of *V. cholerae* strains were analyzed for GQ motif prediction by utilizing G-tract = 3 and loop length 1–20 using our in-house developed GQ prediction tool (Version 2.0) (Mishra et al., 2016). A total of 68 complete genomes available in NCBI Genome database till January 31, 2020 were analyzed for GQ prediction (Supplementary Table 1). G-quadruplex prediction analysis revealed an average ∼85 G-quadruplex forming motifs (VC-PGQs) in chromosome I and ∼45 PGQs in chromosome II (Supplementary Dataset S2). For the reference genome, *V. cholerae* O1 biovar El Tor str. N16961 strain, 86 PGQs with 43 each in a sense, and anti-sense strands were observed for chromosome I (Figure 1D and Supplementary Dataset 2). Out of these, four PGQs in the sense strand and seven PGQs in the anti-sense strands were observed in the promoter regions of various genes while the rest were observed to be located in the open reading frames. Similarly, for chromosome II, 17 PGQs (1 in the promoter region, 16 in ORFs) in the sense and 26 PGQs (7 in promoter and 19 in ORFs) in the anti-sense strands were predicted that satisfied the GQ prediction parameters (Figure 1D and Supplementary Dataset 2). Further sorting on the basis of conservation, ten unique PGQs, seven in chromosome I and three in chromosome II, were retrieved with maximum percentage of conservation among all the available genomes (Table 1 and Supplementary Figure 1). These conserved motifs were also predicted using QGRS Mapper that provided a good G-score that strengthens the propensity of GQ formation by these motifs (Supplementary Table 2).

**TABLE 1:**
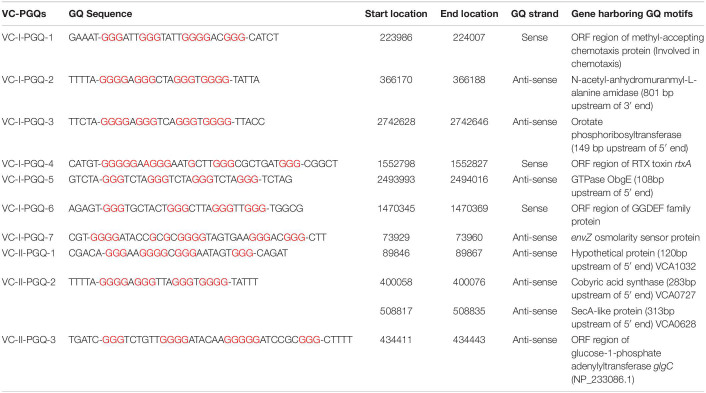
List of 10 conserved VC-PGQs along with their locations, strands, and genes harboring them in chromosome I (NC_002505.1) and II (NC_002506.1) in the reference genome of *Vibrio cholerae.*

*Five bases are added to the GQ motifs and these sequences along with upstream and downstream bases were used for biophysical studies. The guanines participating in the G-quartet formation are colored red.*

### NMR Spectroscopy Displays the Distinctive Imino Peaks of the GQ Specific Hoogsteen Bonds in VC-PGQs

**FIGURE 2:**
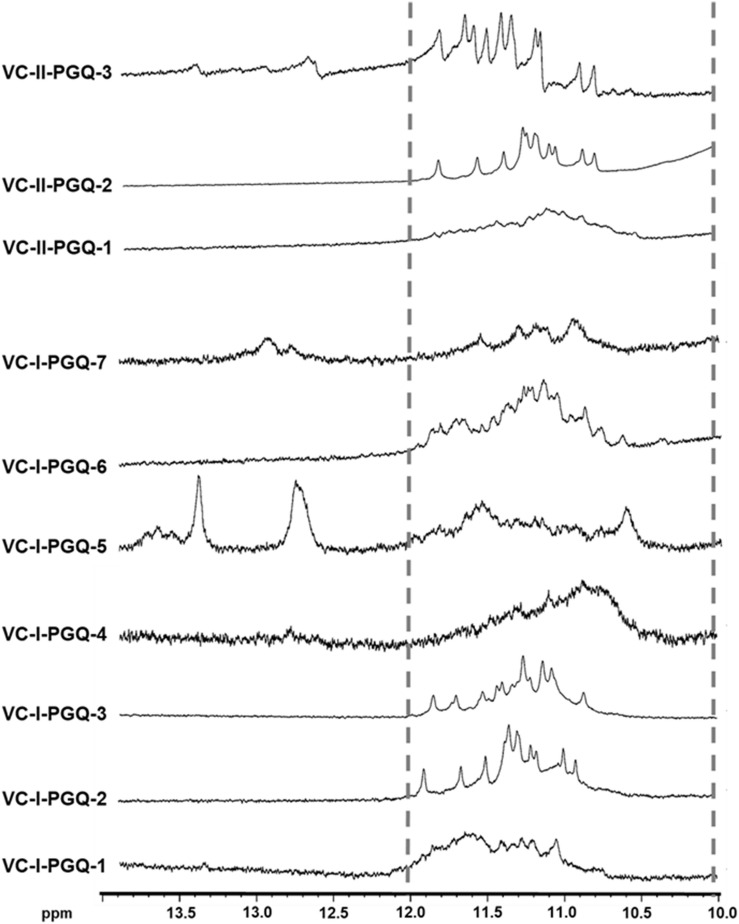
^1^H NMR Spectroscopy of VC-PGQs. NMR spectra obtained for the seven PGQs of chromosome I and II of *V. cholerae*. All the VC-PGQs showed the peaks in the amino region (10–12 ppm, shown by dotted line).

Nuclear Magnetic Resonance spectroscopy is one of the most quintessential techniques used to explore GQ formation. The 1D proton NMR spectra performed for the VC-PGQ sequences dissolved in the potassium containing buffer revealed the distinctive chemical shift signal in the 10–12 ppm region which showcases the imino protons involved in the Hoogsteen hydrogen bond formation of a GQ structure (Feigon et al., 1995; Figure 2). The GQ structures are formed by stacking of G-quartets, which are in turn made up of four guanine residues interconnected by the help of Hoogsteen bonds. These Hoogsteen hydrogen bonds differ from the canonical hydrogen bonds involved in the Watson-Crick base pairing as they link the amino and imino protons of one guanine base to the nitrogen (N7) and oxygen (O6) atoms of the adjacent guanine, respectively. The imino protons involved in the G-tetrad formation are very stable and exchange very slowly with the solvent molecules, thereby allowing their detection through the NMR technique for a long duration of time (Adrian et al., 2012). All the ten VC-PGQs present in both *V. cholerae* chromosomes clearly displayed the peaks in the region between 10 and 12 ppm, which established the fact that the predicted VC-PGQs form stable GQ structures (Figure 2). Along with the GQ chemical shift, three PGQs, VC-I-PGQ-5, VC-I-PGQ-7, and VC-II-PGQ-3, also revealed the formation of small peaks in the 12-14 ppm region depicting the formation of an additional secondary structure with canonical Watson-crick bonding along with the GQ conformation (Figure 2). Presence of a chemical shift in both the regions simultaneously depicts the presence of GQ-hairpin-duplex conformation similar to the one observed in *Escherichia coli* (Kaplan et al., 2016).

### Prediction of the Topology Exhibited by VC-PGQs Structure by Circular Dichroism Spectroscopy in the Presence of Various Cations

**FIGURE 3:**
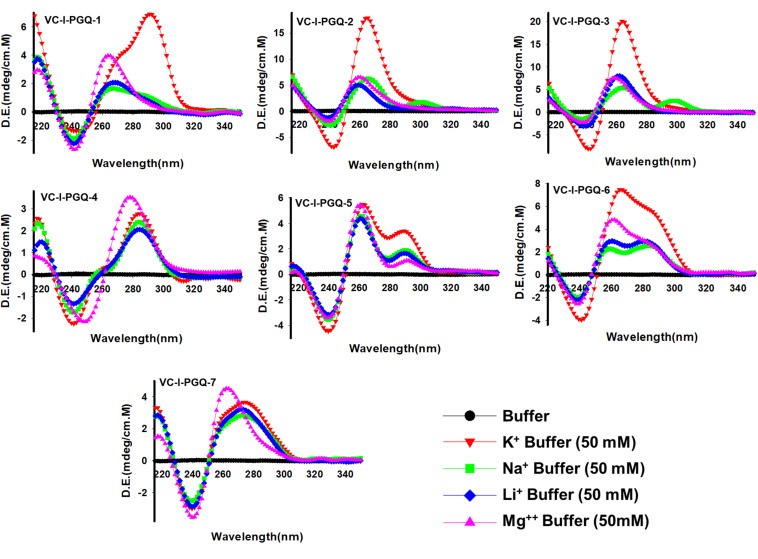
Circular Dichroism spectral analysis of chromosome I in the presence of various cations. CD spectra obtained for VC-PGQ-1-7 of chromosome I in the presence of 50 mM K^+^, Na^+^, Li^+^, or Mg^2+^.

**FIGURE 4:**
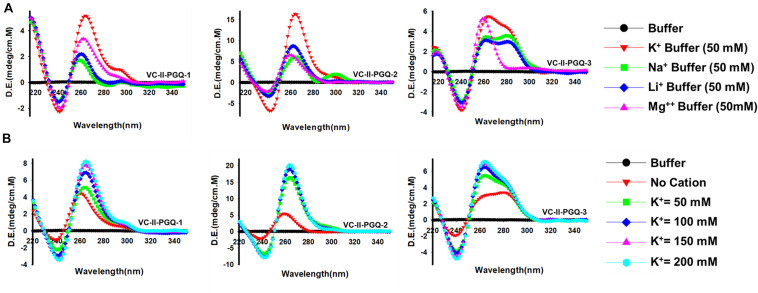
Circular Dichroism spectral analysis of VC-PGQs of Chromosome II. **(A)** CD Spectra obtained for VC-II-PGQ-1-3 in 50 mM of K^+^, Na+, Li^+^, and Mg^2+^. **(B)** CD spectra obtained by increasing concentrations of K^+^ (from 0 to 200 mM) for VC-II-PGQs. For the CD spectra of VC-I-PGQs with increasing concentrations of K^+^, please see Supplementary Figure 2.

The GQ structures demonstrate structural polymorphism due to factors such as their sequence composition and loop length physiological environment. Preliminary ideas regarding the structural conformation of the GQ structures can be obtained through the Circular Dichroism spectroscopy, as it provides us with the signature pattern of spectra related to the relative strand orientation and folding during the GQ formation in a particular physiological condition. A large body of evidence suggests that parallel topology of GQ is confirmed when a positive peak at ∼265 nm and a negative peak at ∼240 nm is observed in the CD spectra whereas the anti-parallel topology is indicated when the positive peak appears in ∼290 nm and the negative peak at the ∼260 nm. More often, a hybrid or mixed topology may arise during GQ formation exhibiting two simultaneous positive peaks at 260nm and 290 nm with a single negative peak at 240 nm (Kypr et al., 2009; Vorlíčková et al., 2012). Evaluation of VC-PGQs in the presence of different cations through CD spectroscopy revealed that in the presence of K^+^ ion, all VC-PGQs other than VC-I-PGQ-2 and VC-I-PGQ-3 showed mixed/hybrid topology while the latter two exhibited parallel topology with a single sharp positive peak at 260nm (Figures 3, 4A). Mixed topology in the VC-I-PGQ-1 and VC-I-PGQ-4 depicted the predominance of anti-parallel topology in the presence of 50 mM K^+^ ion and a small population of parallel GQ conformation. Simultaneously in the presence of Na^+^ containing buffer, all the VC-PGQs displayed the hybrid topology. Potassium and sodium are largely known cations that stabilize the GQ structures but Magnesium (Mg^2+^), a divalent cation, and Lithium (Li^+^) show less or neutral effect on the GQ formation which is evident from the CD spectra results obtained for all the VC-PGQs (Bhattacharyya et al., 2016; Figures 3, 4A).

We moved a step ahead on evaluating the CD spectra pattern on the increasing concentration of potassium ion, and we observed a distinct increase in the peak intensity with the increasing concentration of K^+^ ion in all the sequences (Figure 4B and Supplementary Figure 2). While in most cases only the peak intensity was increased on the addition of K^+^ ions due to higher stabilization of the GQ structures, in the case of VC-I-PGQ-7, a transition from hybrid topology at K^+^ = 0 mM to the parallel topology at K^+^ = 50 mM (Supplementary Figure 2) and for VC-II-PGQ-2, a conversion from the parallel topology at K^+^ = 0 mM to hybrid topology at K^+^ = 50 mM, were noticed (Figure 4B).

**FIGURE 5:**
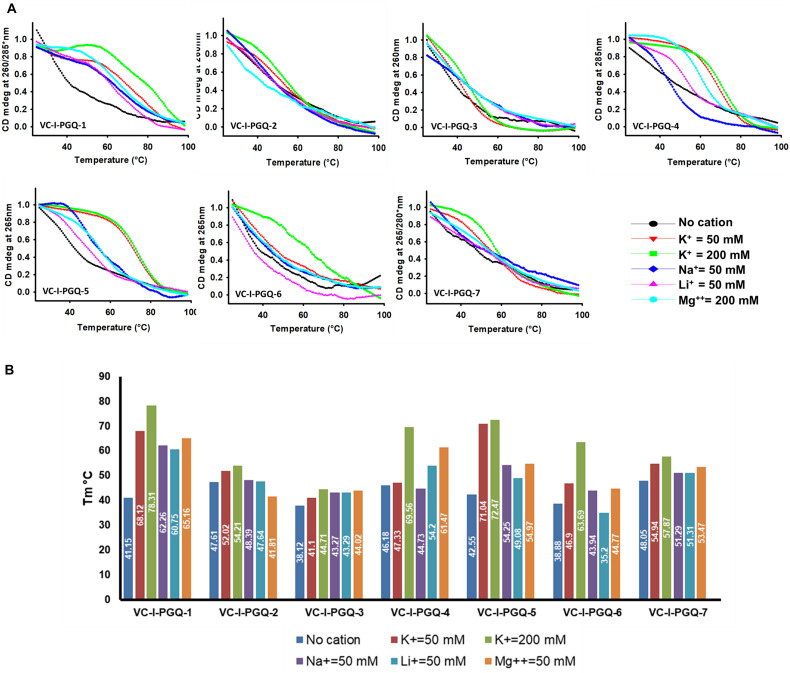
Circular Dichroism Melting analysis of VC-PGQs in Chromosome I. **(A)** Melting curves obtained by heating VC-I-PGQs in the absence and/or presence of various cations. **(B)** Bar graph representing the T_m_ of VC-I-PGQs in various thermal conditions (For Chromosome II melting data, please see Supplementary Figure 3).

On analyzing the CD melting graph of the VC-PGQs, a clear picture of the stability of the GQ structures were obtained. In all the VC-PGQs, the presence of K^+^ predominantly revealed better stabilization and a higher melting temperature value (Tm) as compared to Na^+^, Li^+^, and Mg^2+^ (Figure 5 and Supplementary Figure 2). The Tm even rises higher when the potassium concentration is increased from 50 to 200 mM in all cases. A distinct difference in Tm value is observed when cation is absent and when cation is present; in the case of VC-I-PGQ-1, for example, the Tm value when no cation is present is 41.15 °C while it shoots up to as high as 78.31 °C when the K^+^ concentration is 200 mM.

### Molecularity of the VC-PGQs Evaluated Through Electrophoretic Mobility Shift Assay

Gel electrophoresis helps in resolving DNA or RNA oligonucleotides according to their molecular size, and therefore this technique is useful for assessing the molecularity of the GQ structures. G-quadruplex structures are predominantly formed by folding of the interconnection of Guanine residues in one single strand of DNA or RNA forming the intra-molecular GQ structures, but Guanine residues from more than one strand can assemble together to give rise to inter-molecular GQ structures (Figures 1B,C). Thus, when these GQ sequences are evaluated through gel electrophoresis, their mobility is different from their linear counterparts. The intra-molecular GQ structures forming the compact structures run faster while the inter-molecular GQ structures run slower due to the association of multiple strands in comparison to the non-GQ forming sequences of the same length (Víglaský et al., 2010). Interestingly, all the VC-PGQs of both chromosomes show faster mobility and, therefore, are visibly ahead of their linear counterparts (Supplementary Figure 4). The similar band mobility shift was observed in the positive control, *cKit22*, as compared to its linear counterpart. Thus, all of them form the intra-molecular GQ structures, which are preferable for the stability and propensity of the GQ formation. In certain cases, like VC-I-PGQ-2, VC-I-PGQ-3, and VC-I-PGQ-7, we observed the appearance of two bands, which may be attributed to the formation of two populations of GQ with different conformations (Supplementary Figure 4).

### Strong Binding Affinity of VC-PGQs With the Known GQ Binding Ligand, TMPyP4

**FIGURE 6:**
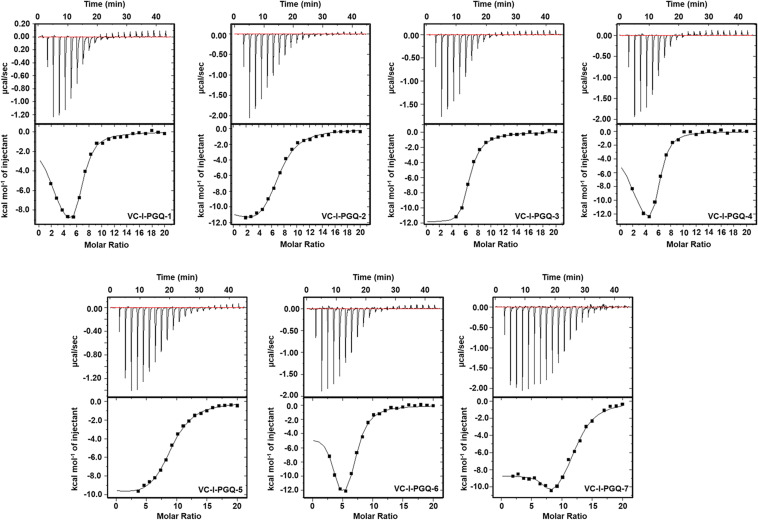
Thermograms obtained by Isothermal Calorimetry obtained for the interaction of TMPyP4 with VC-I-PGQs.

**FIGURE 7:**
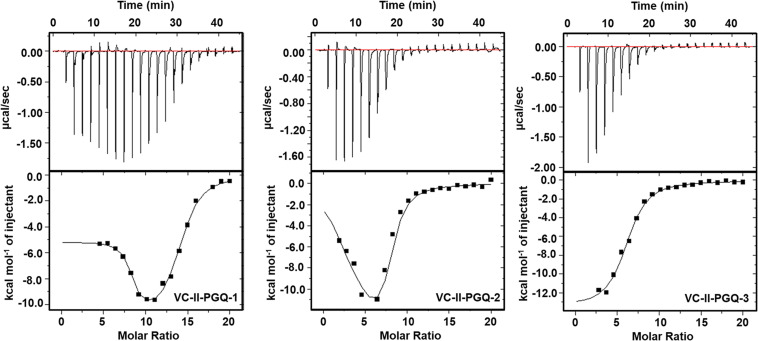
Thermograms obtained by Isothermal Calorimetry obtained for the interaction of TMPyP4 with VC-II-PGQs.

GQ structures have been explored as potential drug targets in recent decades for a number of diseases including cancer and viral and bacterial diseases. Parallelly, the development of GQ targeting ligands have also increased in pace, with molecules like Quarfloxin (targeting the telomeric GQ structures) reaching the clinical trial phase (Yang and Okamoto, 2010). A number of GQ specific ligands, like TMPyP4, Braco-19, and Pyridostatin, specifically bind to GQ structures over the duplex DNA and therefore are extensively used to check the binding ability of the GQ structures (Ruggiero and Richter, 2018). In our study we have used TMPyP4 molecules to evaluate the propensity of the GQ structures to bind with the GQ binders. TMPyP4 is a cationic porphyrin molecule that can stack and stabilize the GQ structures due to its intrinsic properties (Parkinson et al., 2007). Isothermal Calorimetric Titration technique was utilized to study the binding affinity of the VC-PGQs with the TMPyP4 molecule as it is one of the most reliable, sensitive, and accurate techniques commonly used for studying biomolecular interaction and provides the complete thermodynamic profile in one go (Funke and Weisz, 2019). ITC studies revealed that all the VC-PGQs possessed a strong binding affinity towards TMPyP4 as the enthalpograms show the proper binding curves (Figures 6, 7). The thermodynamic reaction for all the PGQ sequences gave negative enthalpy values that indicate the energetically favorable interaction with the TMPyP4 molecule. VC-II-PGQ-1 had the highest binding affinity towards TMPyP4 with the K_d_ value being 0.22 μM, followed by VC-I-PGQ-7 having a K_d_ value of 0.1 μM. These two VC-PGQs were further evaluated by molecular dynamics studies. The other VC-PGQs also provided significant binding affinity values with negative enthalpic energy, as mentioned in Supplementary Table 3.

### Molecular Docking Simulation Revealed the Molecular Interaction of TMPyP4 With the VC-PGQs

**FIGURE 8:**
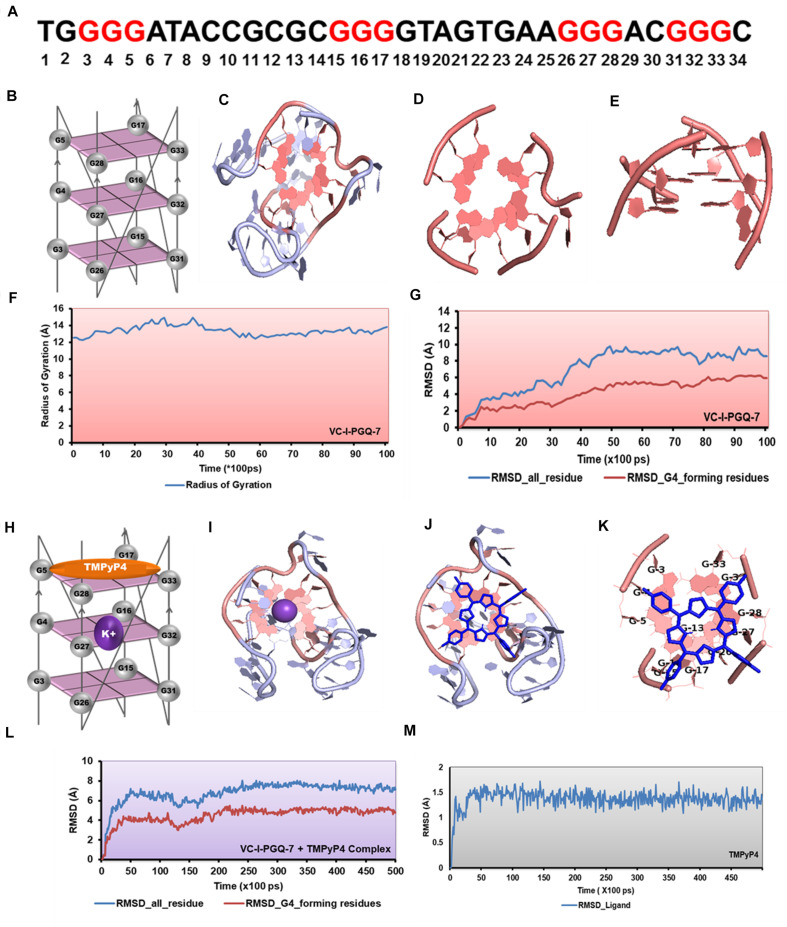
Molecular docking and simulation analysis of VC-I-PGQ-7. **(A)** VC-I-PGQ-7 GQ sequence along with the highlight G that participates in the tetrad formation in the modeled structure. **(B)** Diagrammatic representation of the modeled structure depicting the G-tetrad formation with the participating Guanine residues. **(C)** Representation of the modeled structure in cartoon form. **(D,E)** Figures depicting the stacking of G-tetrads in the G-quadruplex conformation. **(F,G)** Radius of gyration (Rg) and RMSD plots obtained w.r.t. to time frames showing the compactness and stability of the modeled structure. **(H)** Cartoon representation of VC-I-PGQ-7 along with the binding site of TMPyP4 and K^+^ ion. **(I)** Interaction of K^+^ in the center of the G-tetrads. **(J,K)** Structures depicting the docked complex of VC-I-PGQ-7 with TMPyP4. **(L,M)** RMSD of the complex and ligand obtained from a 50 ns simulation analysis.

**FIGURE 9:**
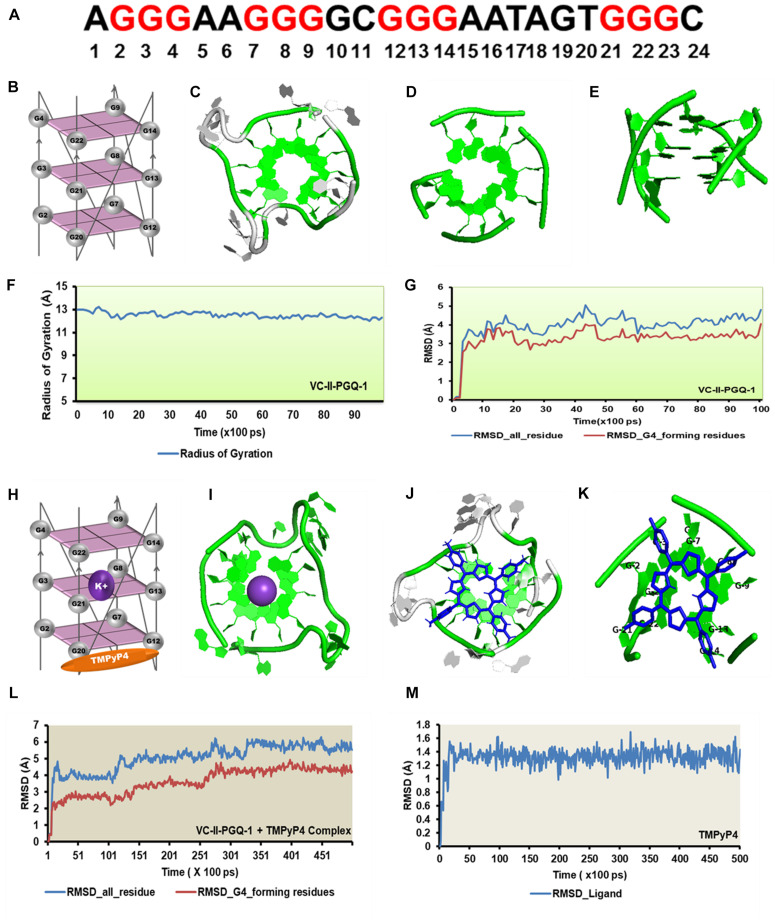
Molecular docking and simulation analysis of VC-II-PGQ-1. **(A)** VC-II-PGQ-1 GQ sequence along the highlight G that participates in the tetrad formation. **(B)** Diagrammatic representation of the modeled structure depicting the G-tetrad formation with the participating Guanine residues. **(C)** Representation of the modeled structure in cartoon form. **(D,E)** Stacking of G-tetrads in the G-quadruplex conformation. **(F,G)** Radius of gyration (Rg) and RMSD plots obtained w.r.t. to time frames showing the compactness and stability of the modeled structure. **(H)** Cartoon representation of VC-II-PGQ-1 along with the binding site of TMPyP4 and K^+^ ion. **(I)** Interaction of K^+^ in the center of the G-tetrads. **(J,K)** Structures depicting the docked complex of VC-II-PGQ-1 with TMPyP4. **(L,M)** RMSD of the complex and ligand obtained from a 50 ns simulation analysis.

Circular Dichroism analysis revealed the strong interaction of VC-PGQs with potassium cation that leads to their stabilization. Similarly, ITC analysis revealed the strong interaction of TMPyP4 with the GQs. To gain further insight into the molecular interaction of potassium and TMPyP4 with the *V. cholerae* G-quadruplexes, VC-I-PGQ-7 and VC-II-PGQ-1 were selected as they showed the highest binding affinity with TMPyP4 in the ITC experiments. First, their structures were modeled and then equilibrated and simulated for 10 ns (Figures 8, 9). The modeled structures of VC-I-PGQ-7 (Figures 8A–E) and VC-II-PGQ-1 (Figures 9A–E) showed the characteristic tetrad formation and tetrad stacking, further affirming the formation of GQ conformations by these PGQs. The global fluctuations analysis revealed the initial fluctuations in the modeled GQs till 200 ps, which thereafter remained constant throughout the production run of 10 ns (Figures 8G, 9G). Radius of gyration, another factor for analyzing the stability of the modeled structures that depicts the global compactness of the system, was also analyzed during the simulation analysis (Figures 8F, 9F). The minimal fluctuations in the Rg further affirms the stability of the modeled structures. On comparing the global fluctuations in the whole molecule as compared to that of the Guanine residues participating in G-tetrad and stacking formation, it was observed that the tetrad guanine showed lesser fluctuations as compared to the whole macromolecule (Figures 8G, 9G). This supports the strong interaction of the Guanines of G-tetrad by classical Watson and crick and Hoogsteen hydrogen bonds.

Interaction analysis of K^+^ cation with the VC-I-PGQ-7 and VC-II-PGQ-1 revealed the binding of the cation at the middle of the GQ. It is fitted between the 1 and 2 tetrads in both VC-PGQs (Figures 8H–I). This affirms the hypothesis of stabilization of GQ conformations by various cations where the cations fit between the tetrad neutralizes the negative charges, thereby leading to stabilization of the PGQs. The docking analysis of VC-I-PGQ-7 with TMPyP4 revealed a favorable interaction with a binding energy of −7.43 kcal/mol and predicted inhibitory constant (K_i_) of 3.57 μM. Structural analysis of the docked complex revealed the stacking of TMPyP4 on the bottom tetrad. Molecular simulation analysis revealed the stable VC-I-PGQ-7 – TMPyP4 complex formation with minimal global fluctuations in the GQs and the ligand throughout the 50 ns simulation (Figures 8L,M).

Similarly, TMPyP4 binds with VC-II-PGQ-1 with a binding energy of −7.44 kcal/mol and the predicted K_i_ of 3.52 μM, and the ligand stacks upon the upper tetrad and forms a stable complex which was supported by the molecular dynamics simulation analysis (Figure 9). The constant RMSD of GQ and TMPyP4 through the trajectory depicted the stable and energy favorable complex formation between the GQs and ligands (Figures 9L,M).

## Discussion

*Vibrio cholerae* is an autochthonous inhabitant of aquatic systems, often causing an acute life-threatening diarrheal condition termed cholera (Reidl and Klose, 2002). The bacteria have caused seven pandemics till date, with the first six pandemics between 1899 and 1923 caused by the classical biotype of O1strain, while the latest (which has been ongoing since 1961) is being attributed to the El Tor strain. *V. cholerae* possess a unique bipartite genome, i.e., that whole genetic information is coded in two chromosomes (Escudero and Mazel, 2017). This distinctive feature probably provides the bacteria with the added advantage of a shorter replication time and, thereby, shorter doubling time. Among the two chromosomes, chromosome I is the larger one being ∼3 Mb long and coding for the characterized genes possessing essential roles in cellular functioning including DNA replication, transcription, translation, cellular metabolism, and biosynthesis of the cell wall and those involved the bacterial pathogenesis. In contrast, chromosome II is small chromosome of ∼1 Mb in length with a greater proportion of the uncharacterized genes. Chromosome II is believed to have been acquired from a large plasmid by the *Vibrio* family during evolution (Heidelberg et al., 2000). The virulence of *V. cholerae* is attributed to a number of factors which include cholera toxin, toxin-coregulated pilus (TCP) that helps in intestinal colonization, *O*-antigen of Lipopolysaccharides, the bacteria’s motility accommodated by the polar flagellum, porins, and some other unrecognized genes (Klose, 2001). The *V. cholerae* genome has been constantly evolving with the changes in the environmental conditions, and the *V. cholerae*’s mobilome is majorly responsible for its adaptation (Banerjee et al., 2014). Horizontal gene transfer, gene-capturing systems termed integrons, and other mobile genetic elements encompassing the transposons, bacteriophages, conjugative plasmids, ICEs, and SXT elements contribute to most the gene adaptation by the bacteria (Mooi and Bik, 1997; Cho et al., 2010). With changes in genetic constituent, the bacteria has also developed drug resistance, and even multi-drug resistant strains have emerged. Along with the mobile genetic elements, efflux pumps and spontaneous chromosomal mutations are a significant cause behind the antibiotic resistance mechanism (Kitaoka et al., 2011a). Therefore, to curb this bacterial infection, a conserved motif is essential to act as a potential drug target, and GQ structure forming sequences has proven to be one of the ideal motifs in this respect. G-quadruplex motifs, depending upon its location, regulates the expression of the genes. PGQ motifs at the upstream/promoter region of the gene are reported to regulate the transcription of the harboring genes and are widely used as therapeutic targets against various pathogenic viruses, bacteria, protozoa, and other human diseases including cancer and neurological disorders (Balasubramanian et al., 2011; Kota et al., 2015; Tosoni et al., 2015; Perrone et al., 2017a; Shankar et al., 2020). Stabilization of PGQ motifs in the open reading frame of a gene has been reported to suppress their expression (Endoh et al., 2013b). For example, GQ motif in the ORF region of human estrogen receptor alpha regulates its translation (Endoh et al., 2013a). Similarly, stabilization of GQ motif causes an unusual −1 ribosomal frameshift in mammalian cells (Endoh and Sugimoto, 2013). G-quadruplex motifs in the C9orf72 are responsible for the blockade of various functional proteins responsible for neurodegenerative diseases and are one of the main causes of Amyotrophic lateral sclerosis and frontotemporal dementia (Wang et al., 2019). In bacterial species, the presence of GQ motifs in the open reading frame have been shown to have an impeding effect on the transcription of the PGQ harboring genes (Mishra et al., 2019a, b). The GQ formed in either strands reduces the translation efficiency by either impeding the transcriptional machinery or translational machinery. The difference in the transcriptional or translational effect varies with the position of the PGQ in the sense or anti-sense strand. The PGQ motifs in the sense strand inhibit the transcriptional machinery while in the anti-sense strand of the ORF region the motifs lead to modulation in translation (Agarwal et al., 2014; Holder and Hartig, 2014; Armas et al., 2017). As these GQs impede the formation of mRNA, various strategies have been used for blocking the formation of these GQ motifs in the essential genes of the cells (Armas et al., 2017). Hence, the subsequent stabilization of the PGQs at the upstream region or in the ORF region of sense or anti-sense strands by the G4 specific ligands can be used as a potential therapeutic approach for inhibiting the expression of the desired PGQ harboring genes. With this hypothesis, the genome of the *V. cholerae* was screened thoroughly for the presence of conserved GQ motifs.

Through our genome-wide search of the recent pandemic-causing *V. cholerae* strain, O1 biovar El Tor str. N16961, we observed ten of the most conserved VC-PGQ forming sequences across both the chromosomes of the bacteria. These PGQs were conserved in all the available strains of *V. Cholerae*, including MDR and XDR strains. The most extensively studied multi-drug resistant strain, O1 serotype str. 2010EL-1786, harbors nine out of ten conserved VC-PGQs (Supplementary Table 4). In chromosome I, two of the sequences, VC-I-PGQ-1 and VC-II-PGQ-3, were found to be conserved in the ORF of the methyl-accepting chemotaxis proteins (MCPs). Chemotaxis is an essential property of many bacteria, including *V. cholerae*, possibly contributing to the virulence and survival factor of the bacteria. This phenomenon of bacterial motility in response to environmental stimuli involves a cascade of signal transduction processes in which the MCPs are an integral part. The membrane scanning MCPs receives the signal for the chemoeffectors and thereafter undergo a conformational change due to binding of an attractant or leaving of a repellent. This change in the MCPs is detected by the CheA–CheW complex and, consequently, autophosphorylates the CheA protein kinase, and the subsequent phosphate is passed on to the response regulator, CheY. The phosphorylated CheY ultimately controls the flagellar motor that results in the reorientation of the bacterial cell movement (Boin et al., 2004). While VC-I-PGQ-4 is harbored in the ORF of the important gene, *rtxA* codes for another exotoxin with leukotoxic, hemolytic, and leukocyte-stimulating activities (Lin et al., 1999). ORF regions of two other important genes coding GGDEF family protein, acting as diguanylate cyclase involved in the nucleotide cyclization process and osmolality sensor protein, possess the VC-I-PGQ-6 and VC-I-PGQ-7 sequences, respectively. Certain VC-PGQs were also found in the promoter regions of certain genes coding for Orotate phosphoribosyltransferase, the essential enzyme for pyrimidine biosynthesis, ObgE GTPase, and cobyric acid synthase (Table 1). The VC-I-PGQ-3, VC-I-PGQ-5, VC-II-PGQ-1, and VC-II-PGQ-2 located in the gene promoters may have specialized roles to play in the bacterial gene modulation which need to be analyzed further. Additionally, VC-II-PGQ-2 is present at multiple locations in *V. cholerae* and is also conserved in various human pathogenic bacterial species including *E. coli*, *K. pneumoniae*, *Yersinia pestis*, *Shigella flexineri*, *Salmonella enterica*, *Pseudomonas aeruginosa*, and *M. tuberculosis* (Kaplan et al., 2016). The presence of this PGQ motif in human pathogens strengthens the plausibility of its essential role in the host pathogenesis.

All the biophysical techniques, including NMR, CD, and EMSA, portrayed that the predicted VC-PGQs have the potential to form stable GQ structures and even have the tendency to bind specifically to the GQ binding ligand, TMPyP4, thereby opening up new hopes for the treatment of this prolonged bacterial infection. The molecular dynamic studies helped us to get a better insight regarding the GQ orientation and their interaction pattern with TMPyP4, which also depicted the stability of the GQ-ligand complexes. The present study provides the preliminary base as of how non-canonical secondary nucleic acid structures can bear crucial gene modulatory roles in *V. cholerae* and should be further validated in the real bacterial paradigm. Therefore, other than the conventional drug targets of *V. cholerae*, such as the signaling and transport proteins, toxins protein, ion channel regulators, etc., conserved GQ structure in the *V. cholerae* genome could rise as an alternate therapeutic solution.

## Data Availability Statement

All datasets presented in this study are included in the article/Supplementary Material.

## Author Contributions

AK designed the data conceptualization and methodology. US and NJ performed the *in silico* and *in vitro* experiments. AK, US, PM, and NJ collectively wrote the manuscript. PK, TKS, and AK did the critical review and editing. All authors contributed to the article and approved the submitted version.

## Conflict of Interest

The authors declare that the research was conducted in the absence of any commercial or financial relationships that could be construed as a potential conflict of interest.

## Footnotes

1 http://www.ncbi.nlm.nih.gov/genomes
