# Supplementary material for: Exploring Computational and Biophysical Tools to Study the Presence of G-Quadruplex Structures: A Promising Therapeutic Solution for Drug-Resistant Vibrio cholerae
Source: Front Genet. 2020 Sep 25;11:935. doi: 10.3389/fgene.2020.00935 (PMC7545536; doi:10.3389/fgene.2020.00935)
Supplement: Supplementary file 1 [file Data_Sheet_1.docx]

Supplementary Material

# Data sheet 1: Supplementary Figures S1-S4 and Tables S1-S4.

# Supplementary Figures and Tables

## Supplementary Figure S1: Consensus logo representation of conserved G-quadruplex motifs in the chromosome I and II of *Vibrio cholerae* strain.


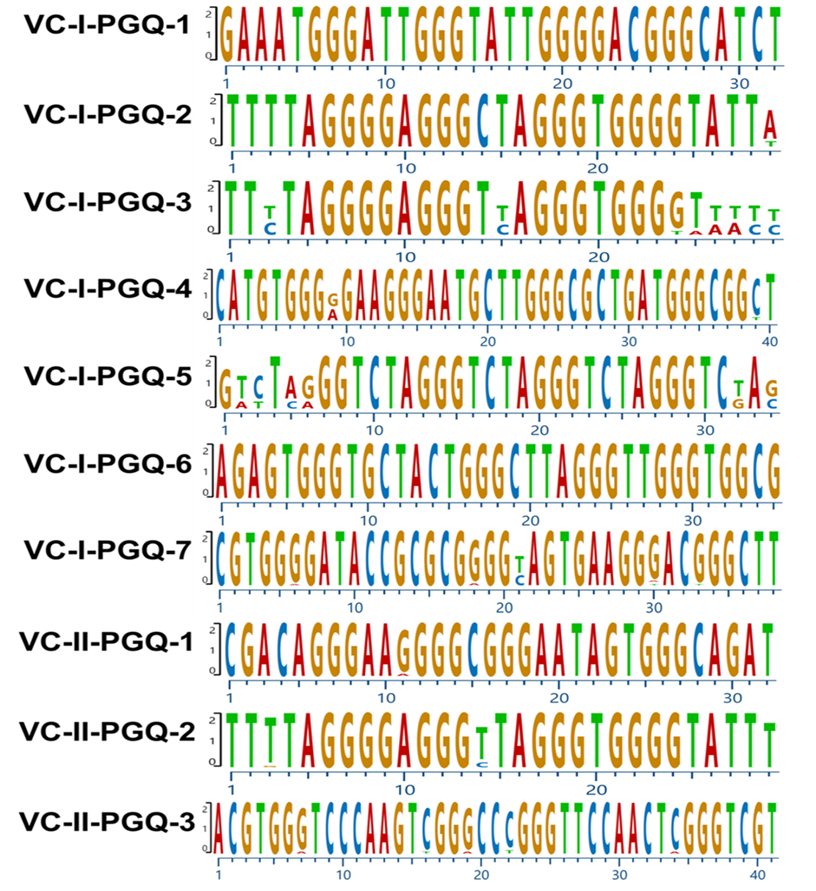


**1.2 Supplementary Figure S2:** **Circular Dichroism spectral analysis of VC-I-PGQs in the presence of increasing concentration of K+ ion (0 to 200 mM).**

**
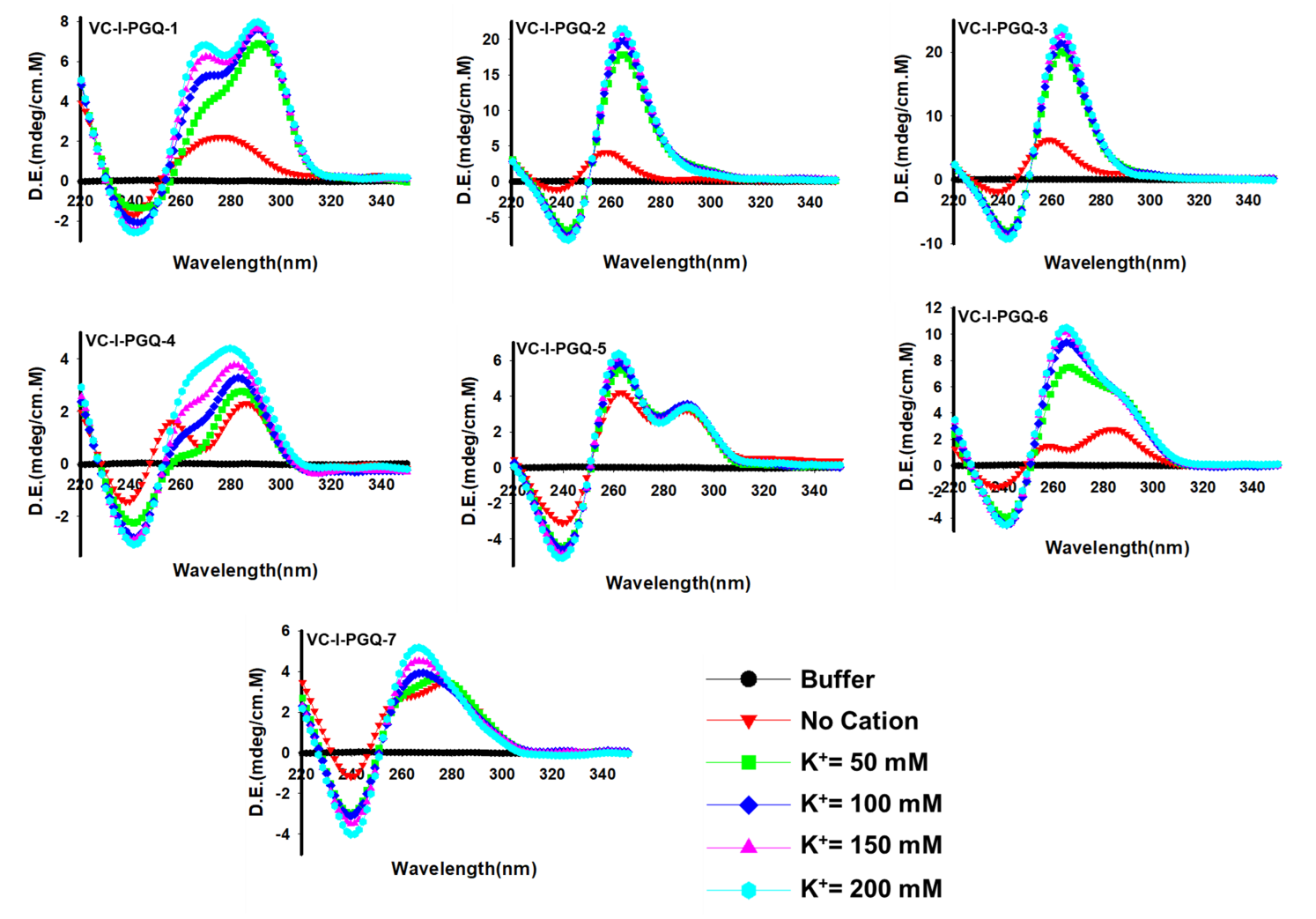
**

**1.3 Supplementary Figure S3:** **Circular Dichroism melting analysis of VC-PGQ motifs in Chromosome II:** (A) Melting curves obtained by heating VC-II-PGQs in the absence and/or presence of various cations. (B) Bar graph representing the Tm of VC-II-PGQs in various thermal conditions.

**
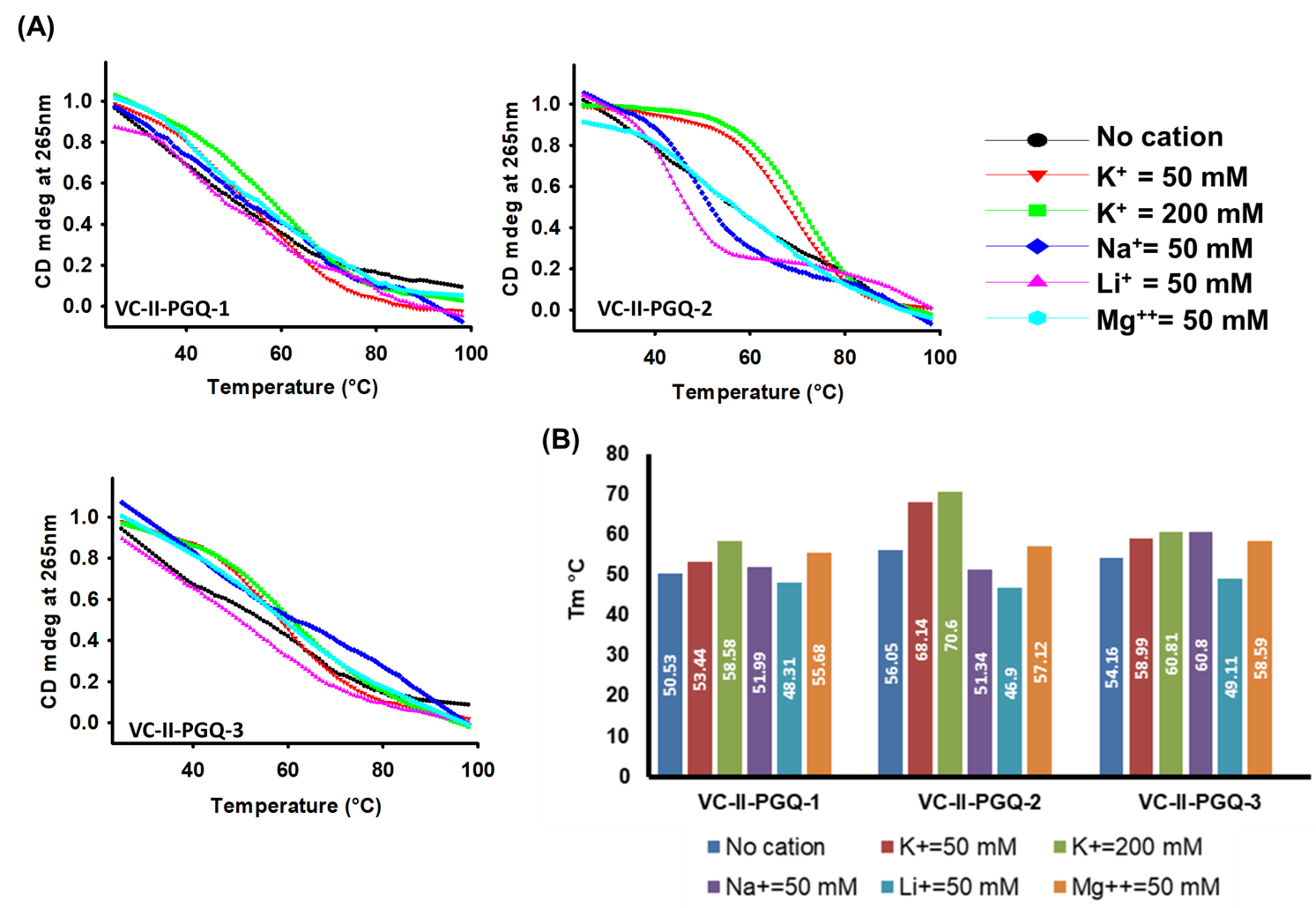
**

**1.4 Supplementary Figure S4:** Results of Electrophoretic mobility shift assay of VC-PGQs in the presence of various cations. *cKit21* and its corresponding mutant of same length were used as positive control. **A:** Mutant of *cKit21*; **B:** *cKit21*; **C:** Mutant of VC-PGQ; **D:** VC-PGQ in K^+^; **E:** VC-PGQ in Na^+^; **F:** VC-PGQ in Li^+^; **G:** VC-PGQ in Mg^2+^; **H:** Mutant of VC-PGQ.


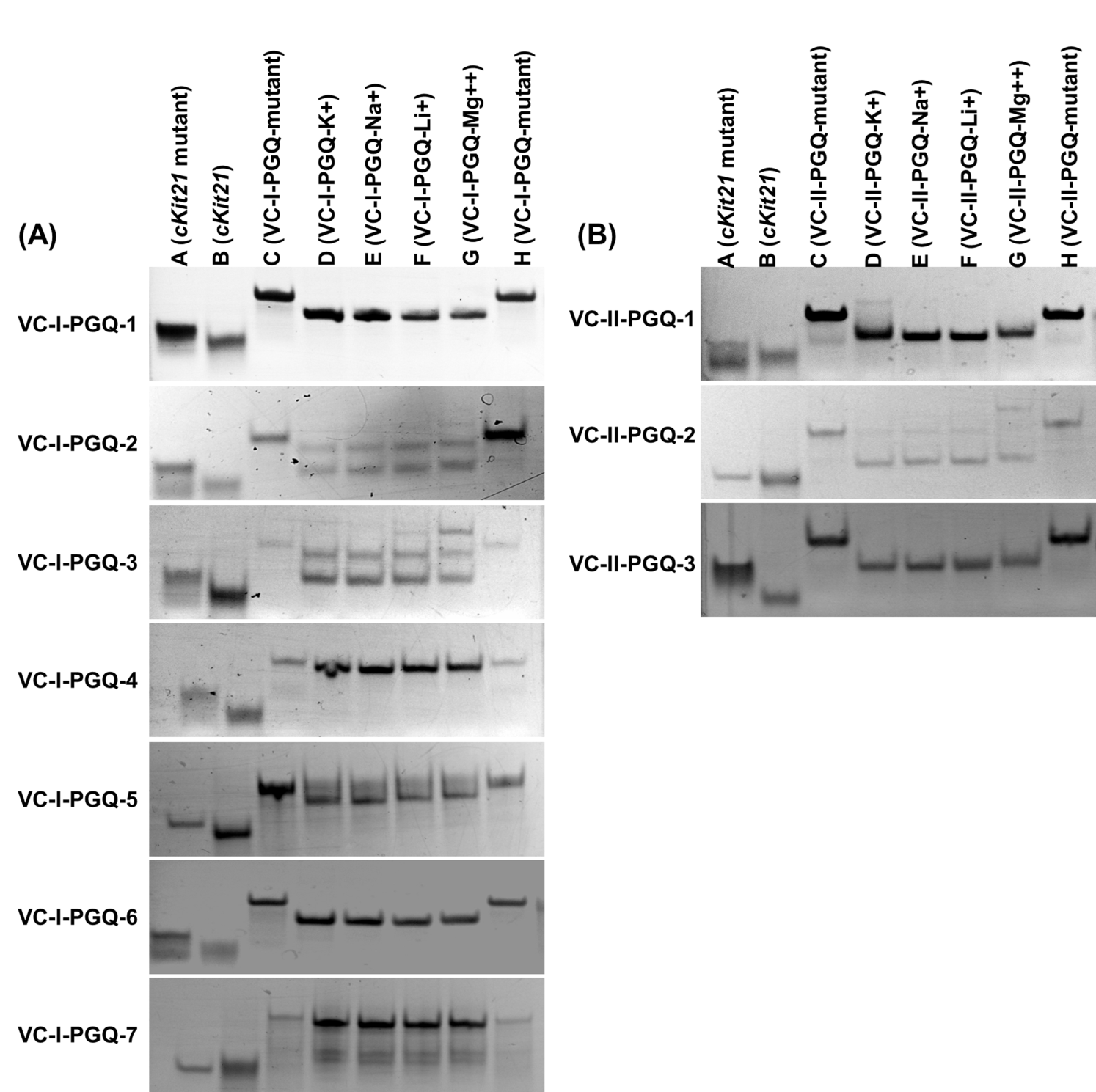


**1.5 Supplementary Table S1:** List of complete genome sequences of *Vibrio cholerae* available at NCBI site that were used for G-quadruplex prediction.

| **#Organism Name** | **Strain** | **Size**  **(Mb)** | **GC%** | **Chromosome I** | **Chromosome II** |
| --- | --- | --- | --- | --- | --- |
| *Vibrio cholerae* | RFB05 | 4.35732 | 47.0257 | NZ_CP043557.1/CP043557.1 | NZ_CP043558.1/CP043558.1 |
| *Vibrio cholerae* | MJ-1236 | 4.23637 | 47.2948 | NC_012668.1/CP001485.1 | NC_012667.1/CP001486.1 |
| *Vibrio cholerae* | 3566-08 | 4.12425 | 47.4507 | NZ_CP046745.1/CP046745.1 | NZ_CP046746.1/CP046746.1 |
| *Vibrio cholerae* | 3528-08 | 4.12654 | 47.4757 | NZ_CP046736.1/CP046736.1 | NZ_CP046735.1/CP046735.1 |
| *Vibrio cholerae* | F9993 | 4.13313 | 47.522 | NZ_CP046840.1/CP046840.1 | NZ_CP046841.1/CP046841.1 |
| *Vibrio cholerae* | E4 | 4.29352 | 47.6975 | NZ_CP033515.1/CP033515.1 | NZ_CP033513.1/CP033513.1 |
| *Vibrio cholerae* | 2011EL-1271 | 4.08146 | 47.7272 | NZ_CP046839.1/CP046839.1 | NZ_CP046838.1/CP046838.1 |
| *Vibrio cholerae* | 3541-04 | 4.12904 | 47.4455 | NZ_CP046747.1/CP046747.1 | NZ_CP046748.1/CP046748.1 |
| *Vibrio cholerae* | 3566-06 | 4.10931 | 47.4721 | NZ_CP046740.1/CP046740.1 | NZ_CP046739.1/CP046739.1 |
| *Vibrio cholerae* | FJ147 | 4.09194 | 47.521 | NZ_CP009042.1/CP009042.1 | NZ_CP009041.1/CP009041.1 |
| *Vibrio cholerae* | 2012EL-2176 | 4.25802 | 47.7124 | NZ_CP007634.1/CP007634.1 | NZ_CP007635.1/CP007635.1 |
| *Vibrio cholerae* | 4295STDY6534232 | 4.09265 | 47.5203 | NZ_LT992488.1/LT992488.1 | NZ_LT992489.1/LT992489.1 |
| *Vibrio cholerae* | 4295STDY6534248 | 4.09264 | 47.5203 | NZ_LT992492.1/LT992492.1 | NZ_LT992493.1/LT992493.1 |
| *Vibrio cholerae* | 4295STDY6534216 | 4.09264 | 47.5203 | NZ_LT992486.1/LT992486.1 | NZ_LT992487.1/LT992487.1 |
| *Vibrio cholerae* | KW3 | 4.08902 | 47.5207 | NZ_CP006947.1/CP006947.1 | NZ_CP006948.1/CP006948.1 |
| *Vibrio cholerae* | 2010EL-1786 | 4.07774 | 47.5204 | NC_016445.1/CP003069.1 | NC_016446.1/CP003070.1 |
| *Vibrio cholerae* | FORC_073 | 4.02609 | 47.6011 | NZ_CP024082.1/CP024082.1 | NZ_CP024083.1/CP024083.1 |
| *Vibrio cholerae* | 4295STDY6534200 | 4.12352 | 47.2082 | LT992490.1 | LT992491.1 |
| *Vibrio cholerae* | FORC_055 | 4.09573 | 47.7612 | NZ_CP016987.1/CP016987.1 | NZ_CP016988.1/CP016988.1 |
| *Vibrio cholerae* | O395 substr. TCP2 | 4.13253 | 47.559 | :NZ_CP045719.1/CP045719.1 | NZ_CP045718.1/CP045718.1 |
| *Vibrio cholerae* | O395 | 4.1353 | 47.5582 | NC_012582.1/CP001235.1 | NC_012583.1/CP001236.1 |
| *Vibrio cholerae* | O395 | 4.13232 | 47.5586 | NC_009456.1/CP000626.1 | NC_009457.1/CP000627.1 |
| *Vibrio cholerae* | C6706 | 4.0903 | 47.4168 | NZ_CP046844.1/CP046844.1 | NZ_CP046845.1/CP046845.1 |
| *Vibrio cholerae* | HC1037 | 4.06142 | 47.5197 | NZ_CP026647.1/CP026647.1 | NZ_CP026648.1/CP026648.1 |
| *Vibrio cholerae* | A1552 | 4.08547 | 47.4904 | NZ_CP028894.1/CP028894.1 | NZ_CP028895.1/CP028895.1 |
| *Vibrio cholerae* | A1552 | 4.08546 | 47.4904 | NZ_CP024867.1/CP024867.1 | NZ_CP024868.1/CP024868.1 |
| *Vibrio cholerae* | A1552 | 4.08546 | 47.4904 | NZ_CP025936.1/CP025936.1 | NZ_CP025937.1/CP025937.1 |
| *Vibrio cholerae* | NCTC9420 | 4.07658 | 47.4904 | NZ_CP013319.1/CP013319.1 | NZ_CP013320.1/CP013320.1 |
| *Vibrio cholerae* | IEC224 | 4.07959 | 47.4898 | NC_016944.1/CP003330.1 | NC_016945.1/CP003331.1 |
| *Vibrio cholerae* | CRC1106 | 4.09912 | 47.4585 | NZ_CP013305.1/CP013305.1 | NZ_CP013306.1/CP013306.1 |
| *Vibrio cholerae* | E1162 | 4.11087 | 47.456 | NZ_CP013309.1/CP013309.1 | NZ_CP013310.1/CP013310.1 |
| *Vibrio cholerae* | C5 | 4.10204 | 47.4574 | NZ_CP013301.1/CP013301.1 | NZ_CP013302.1/CP013302.1 |
| *Vibrio cholerae* | CRC711 | 4.05752 | 47.5164 | NZ_CP013303.1/CP013303.1 | NZ_CP013304.1/CP013304.1 |
| *Vibrio cholerae* | E7946 | 4.06307 | 47.4892 | NZ_CP024162.1/CP024162.1 | NZ_CP024163.1/CP024163.1 |
| *Vibrio cholerae* | 2740-80 | 4.08896 | 47.5305 | NZ_CP016324.1/CP016324.1 | NZ_CP016325.1/CP016325.1 |
| *Vibrio cholerae* | CTMA_1441 | 4.04278 | 47.5641 | NZ_CP047059.1/CP047059.1 | NZ_CP047060.1/CP047060.1 |
| *Vibrio cholerae* | NCTC5395 | 4.17025 | 47.5149 | NZ_CP013317.1/CP013317.1 | NZ_CP013318.1/CP013318.1 |
| *Vibrio cholerae* | BC1071 | 4.19458 | 47.5097 | NZ_LT897797.1/LT897797.1 | NZ_LT897798.1/LT897798.1 |
| *Vibrio cholerae* | N16961 | 4.04784 | 47.4881 | NZ_CP028827.1/CP028827.1 | NZ_CP028828.1/CP028828.1 |
| *Vibrio cholerae* | FDAARGOS_223 | 4.04227 | 47.4878 | NZ_CP020408.2/CP020408.2 | NZ_CP020407.2/CP020407.2 |
| *Vibrio cholerae* | E506 | 4.06251 | 47.5311 | NZ_CP013307.1/CP013307.1 | NZ_CP013308.1/CP013308.1 |
| *Vibrio cholerae* | 3569-08 | 4.05646 | 47.5321 | NZ_CP046744.1/CP046744.1 | NZ_CP046743.1/CP046743.1 |
| *Vibrio cholerae* | FORC_076 | 4.02149 | 47.2842 | NZ_CP026531.1/CP026531.1 | NZ_CP026532.1/CP026532.1 |
| *Vibrio cholerae* | E9120 | 4.06673 | 47.6011 | NZ_CP013313.1/CP013313.1 | NZ_CP013314.1/CP013314.1 |
| *Vibrio cholerae* | A19 | 4.0335 | 47.4873 | NZ_LT907989.1/LT907989.1 | NZ_LT907990.1/LT907990.1 |
| *Vibrio cholerae* | N16961 | 4.0335 | 47.4873 | NZ_LT906614.1/LT906614.1 | NZ_LT906615.1/LT906615.1 |
| *Vibrio cholerae* | N16961 | 4.03346 | 47.4873 | NC_002505.1/AE003852.1 | NC_002506.1/AE003853.1 |
| *Vibrio cholerae* | AAS91 | 4.08571 | 47.6073 | NZ_CP042299.1/CP042299.1 | NZ_CP042300.1/CP042300.1 |
| *Vibrio cholerae* | Sa5Y | 4.05088 | 47.5296 | NZ_CP028892.1/CP028892.1 | NZ_CP028893.1/CP028893.1 |
| *Vibrio cholerae* | E1320 | 4.11044 | 47.518 | :NZ_CP013311.1/CP013311.1 | NZ_CP013312.1/CP013312.1 |
| *Vibrio cholerae* | RFB16 | 4.13841 | 47.4023 | NZ_CP043554.1/CP043554.1 | NZ_CP043556.1/CP043556.1 |
| *Vibrio cholerae* | 20000 | 4.0527 | 47.5356 | NZ_CP036499.1/CP036499.1 | NZ_CP036500.1/CP036500.1 |
| *Vibrio cholerae* | M2140 | 4.01486 | 47.5589 | NZ_CP013315.1/CP013315.1 | NZ_CP013316.1/CP013316.1 |
| *Vibrio cholerae* | 2012Env-9 | 4.06181 | 47.6062 | NZ_CP012997.1/CP012997.1 | NZ_CP012998.1/CP012998.1 |
| *Vibrio cholerae* | MS6 | 4.03094 | 47.4557 | NZ_AP014524.1/AP014524.1 | NZ_AP014525.1/AP014525.1 |
| *Vibrio cholerae* | 2015V-1118 | 4.03067 | 47.6811 | NZ_CP046749.1/CP046749.1 | NZ_CP046750.1/CP046750.1 |
| *Vibrio cholerae* | Env-390 | 4.05093 | 47.6054 | NZ_CP013013.1/CP013013.1 | NZ_CP013014.1/CP013014.1 |
| *Vibrio cholerae* | 3523-03 | 4.11795 | 47.4941 | NZ_CP046742.1/CP046742.1 | NZ_CP046741.1/CP046741.1 |
| *Vibrio cholerae* | NCTC 30 | 3.95236 | 47.2395 | NZ_LS997867.1/LS997867.1 | NZ_LS997868.1/LS997868.1 |
| *Vibrio cholerae* | M66-2 | 3.93891 | 47.5875 | NC_012578.1/CP001233.1 | NC_012580.1/CP001234.1 |
| *Vibrio cholerae* | 2015V-1126 | 4.02047 | 47.6605 | NZ_CP046737.1/CP046737.1 | NZ_CP046738.1/CP046738.1 |
| *Vibrio cholerae* | TSY216 | 5.00049 | 45.6893 | CP007653.1 | CP007654.1 |
| *Vibrio cholerae* | 2011V-1043 | 4.11208 | 47.5166 | CP046837.1 | CP046836.1 |
| *Vibrio cholerae* | K2802 | 3.92396 | 47.7707 | CP046843.1 | CP046842.1 |
| *Vibrio cholerae* | LMA3984-4 | 3.73872 | 47.9734 | CP002555.1 | CP002556.1 |
| *Vibrio cholerae* | 10432-62 | 4.07746 | 47.7 | NZ_CP010812.1/CP010812.1 | NA |
| *Vibrio cholerae* | V060002 | 4.05704 | 47.5 | NZ_AP018677.1/AP018677.1 | NA |
| *Vibrio cholerae* | 1154-74 | 3.92836 | 47.8 | NZ_CP010811.1/CP010811.1 | NA |

**1.6 Supplementary Table S2:** Prediction of the highly conserved ten G-quadruplex motifs of *Vibrio cholerae* using QGRSMapper tool. Guanines participating the G-quadruplex formation as predicted the QGRSMapper are underlined.

| **VC-PGQs** | **Length** | **GQ Sequence** | **QGRSMapper G-Score** |
| --- | --- | --- | --- |
| **Chromosome I** | | | |
| VC-I-PGQ-1 | 22 | GGGATTGGGTATTGGGGACGGG | 71 |
| VC-I-PGQ-2 | 19 | GGGGAGGGCTAGGGTGGGG | 71 |
| VC-I-PGQ-3 | 19 | GGGGAGGGTCAGGGTGGGG | 71 |
| VC-I-PGQ-4 | 30 | GGGGGAAGGGAATGCTTGGGCGCTGATGGG | 69 |
| VC-I-PGQ-5 | 24 | GGGTCTAGGGTCTAGGGTCTAGGG | 72 |
| VC-I-PGQ-6 | 25 | GGGTGCTACTGGGCTTAGGGTTGGG | 67 |
| VC-I-PGQ-7 | 31 | GGGATACCGCGCGGGGTAGTGAAGGGACGGG | 65 |
| **Chromosome II** | | | |
| VC-II-PGQ-1 | 22 | GGGAAGGGGCGGGAATAGTGGG | 68 |
| VC-II-PGQ-2 | 19 | GGGGAGGGTTAGGGTGGGG | 71 |
| VC-II-PGQ-3 | 33 | GGGTCTGTTGGGGATACAAGGGGGATCCGCGGG | 72 |

**1.7 Supplementary Table S3:** List of thermodynamic parameters obtained from Isothermal calorimetric assay used for analyzing the interaction of TMPyP4 with VC-PGQs.

| **VC - PGQs** | **K_1_ (M^-1^)** | **∆H_1_**  **(cal/mol)** | **∆S_1_ (cal/mol/deg)** | **∆G_1_ (kJ)** | **K_2_ (M^-1^)** | **∆H_2_ (cal/mol)** | **∆S_2_ (cal/mol/deg)** | **∆G_2_ (kJ)** |
| --- | --- | --- | --- | --- | --- | --- | --- | --- |
| **Chromosome I** | | | | | | | | |
| **VC-I-PGQ-1** | 6.72E+05 | 4.01E+04 | 1.61E+02 | -7.87E+03 | 5.88E+05 | -6.23E+08 | -2.09E+06 | 6.34E+05 |
| **VC-I-PGQ-2** | 2.45E+05 | 1.42E+07 | 4.76E+04 | -1.94E+03 | 2.31E+05 | -4.75E+04 | -1.35E+02 | -7.23E+03 |
| **VC-I-PGQ-3** | 5.87E+06 | -1.19E+04 | -8.86E+00 | -9.24E+03 | 9.74E+04 | -6.32E+03 | 1.63E+00 | -6.81E+03 |
| **VC-I-PGQ-4** | 8.21E+05 | 5.73E+04 | 2.19E+02 | -8.03E+03 | 9.17E+05 | -4.27E+07 | -1.10E+04 | -3.95E+07 |
| **VC-I-PGQ-5** | 2.34E+05 | 7.04E+04 | 2.61E+02 | -7.42E+03 | 2.23E+05 | -3.27E+04 | -8.51E+01 | -7.32E+03 |
| **VC-I-PGQ-6** | 7.07E+06 | -4.42E+03 | 1.65E+01 | -9.34E+03 | 2.94E+05 | -1.75E+04 | -3.36E+01 | -7.47E+03 |
| **VC-I-PGQ-7** | 9.77E+06 | -8.72E+03 | 2.74E+00 | -9.54E+03 | 1.60E+05 | -1.33E+04 | -2.09E+01 | -7.10E+03 |
| **Chromosome II** | | | | | | | | |
| **VC-II-PGQ-1** | 4.50E+07 | -5.17E+03 | 1.77E+01 | -1.04E+04 | 4.00E+05 | -1.13E+04 | -1.22E+01 | -7.65E+03 |
| **VC-II-PGQ-2** | 8.47E+05 | -1.89E+11 | -6.33E+08 | -1.71E+08 | 8.46E+05 | 9.13E+06 | 3.06E+04 | 5.61E+03 |
| **VC-II-PGQ-3** | 6.16E+07 | -1.48E+04 | -1.41E+01 | -1.06E+04 | 2.93 E5 | -1.22E+00 | -2.50E+01 | 7.45E+03 |

**1.8 Supplementary Table S4:** Location of conserved VC-PGQs in the most extensively studied multi drug resistant Vibrio cholerae O1 str. 2010EL-1786 strain (Accession Number - NC_016445.1 and NC_016446.1). VC-PGQ-5 motif have a single point mutation in the MDR strain that breaks the G-tract.

| **# VC-PGQs** | **Length (bp)** | **Start Position** | **End Position** | **PGQ Motifs (Chromosome I)** | **cG Score** | **cC Score** |
| --- | --- | --- | --- | --- | --- | --- |
| **Chromosome I (NC_016445.1)** | | | | | | |
| **VC-I-PGQ-1** | 22 | 2649254 | 2649275 | **GGGATTGGGTATTGGGGACGGG** | 130 | 10 |
| **VC-I-PGQ-2** | 19 | 966242 | 966260 | **GGGGAGGGCTAGGGTGGGG** | 140 | 10 |
| **VC-I-PGQ-3** | 19 | 387586 | 387604 | **GGGGAGGGTCAGGGTGGGG** | 140 | 10 |
| **VC-I-PGQ-4** | 30 | 1029794 | 1029823 | **GGGGGAAGGGAATGCTTGGGCGCTGATGGG** | 170 | 30 |
| **VC-I-PGQ-5** | 26 | 2891296 | 2891320 | **AGGTCTAGGGTCTAGGGTCTAGGG** |  |  |
| **VC-I-PGQ-6** | 25 | 947338 | 947362 | **GGGTGCTACTGGGCTTAGGGTTGGG** | 130 | 30 |
| **VC-I-PGQ-7** | 32 | 673998 | 674029 | **GGGGATACCGCGCGGGGTAGTGAAGGGACGGG** | 180 | 50 |
| **Chromosome II (NC_016446.1)** | | | | | | |
| **VC-II-PGQ-1** | 22 | 363366 | 363387 | **GGGAAGGGGCGGGAATAGTGGG** | 140 | 10 |
| **VC-II-PGQ-2** | 19 | 673567 | 673585 | **GGGGAGGGTTAGGGTGGGG** | 140 | 0 |
| **VC-II-PGQ-3** | 33 | 707920 | 707952 | **GGGTCTGTTGGGGATACAAGGGGGATCCGCGGG** | 170 | 50 |
